# Hematopoietic Stem and Progenitor Cells as Effectors in Innate Immunity

**DOI:** 10.1155/2012/165107

**Published:** 2012-06-19

**Authors:** Jennifer L. Granick, Scott I. Simon, Dori L. Borjesson

**Affiliations:** ^1^Department of Pathology, Microbiology, Immunology, University of California School of Veterinary Medicine, Davis, CA 95616, USA; ^2^Department of Biomedical Engineering, University of California, Davis, CA 95616, USA

## Abstract

Recent research has shed light on novel functions of hematopoietic stem and progenitor cells (HSPC). While they are critical for maintenance and replenishment of blood cells in the bone marrow, these cells are not limited to the bone marrow compartment and function beyond their role in hematopoiesis. HSPC can leave bone marrow and circulate in peripheral blood and lymph, a process often manipulated therapeutically for the purpose of transplantation. Additionally, these cells preferentially home to extramedullary sites of inflammation where they can differentiate to more mature effector cells. HSPC are susceptible to various pathogens, though they may participate in the innate immune response without being directly infected. They express pattern recognition receptors for detection of endogenous and exogenous danger-associated molecular patterns and respond not only by the formation of daughter cells but can themselves secrete powerful cytokines. This paper summarizes the functional and phenotypic characterization of HSPC, their niche within and outside of the bone marrow, and what is known regarding their role in the innate immune response.

## 1. Introduction

Hematopoietic stem cells (HSC) maintain and replenish all blood cell types in the bone marrow and respond to changing needs for blood cells in peripheral tissues. They give rise to multipotent (produce most blood cell subsets), oligopotent (lymphoid or myeloid restricted), and unipotent hematopoietic progenitor cells (HPC), the latter type restricted to proliferation into a single set of mature blood cells. Throughout embryonic and fetal development, there are multiple sequential sites of hematopoiesis. Though primitive HSC form nucleated red blood cells in the yolk sac, the first definitive HSC appear in the aorta-gonad-mesonephros (AGM) and expand in the fetal liver before finding their niche in the bone marrow (and spleen in rodents) [1, 2]. In adult life, hematopoietic stem and progenitor cells (HSPC) primarily reside in the bone marrow, though recent work has shown that they circulate in blood and lymph and traffic to other hematopoietic and nonhematopoietic organs during homeostasis and stress [3–6]. This roving nature of adult HSPC should not be surprising when one considers the changing location of hematopoiesis throughout HSPC ontogeny. Additionally, extramedullary hematopoiesis is well described in the adult and typically occurs at sites of prenatal hematopoiesis [7]. Given the ability of HSPC to leave the bone marrow and circulate in the periphery, that they express pattern recognition receptors (PRR) and respond to conserved microbial and viral molecular patterns [8], it is reasonable to hypothesize that HSPC are active in innate immune and inflammatory responses outside of the bone marrow.

## 2. Phenotypic and Functional Definitions of Hematopoietic Stem and Progenitor Cells

In order to study the potential function of HSPC outside the bone marrow, it is essential that these cells be discriminated experimentally from leukocytes and other lineage-committed cells. HSPC can be identified by their surface marker expression, unique staining properties of vital dyes, and by specific functional assays. HSC are defined by their ability to self-renew and to produce all blood cell types, while HPC do not have self-renewal capacity and are more restricted in the mature blood cells they can produce. Investigators typically use a combination of functional and phenotypic characteristics to categorize populations of HSPC. 

Fluorescence activated cell sorting (FACS) schemes can identify and enrich stem and progenitor cells in sorted populations, although more committed progenitors are also present. For example, the “KSL” scheme in mice and CD34 in humans identify a heterogenous group of cells including HSC capable of long-term repopulation of bone marrow (long-term repopulating cells or LT-HSC), short-term repopulating cells (ST-HSC), and restricted progenitor cells incapable of long-term hematopoietic reconstitution. Murine KSL cells are c-Kit^+^, Sca-1^+^, and negative for lineage markers of mature blood cell types [9]. The addition of the Flk-2/Flt3 receptor tyrosine kinase to the KSL markers enhances separation of ST-HSC (Flk-2^+^) from LT-HSC (Flk-2^−^) [10]. There is no human homolog for murine Sca-1. Instead, human HSC are identified on the basis of CD34 expression. Interestingly, more primitive HSC in mice have low or absent expression of CD34 [11]. The DNA-binding dye Hoechst 33342 can be used to identify low staining “side populations” (SP) of HSPC [12]. Hoechst staining is often combined with KSL markers to further enrich HSC numbers, so called SP^KLS^ cells. The purity of HSC in sorted SP, KSL or CD34^+^ HSPC can be increased by using the signaling lymphocyte activation molecule (SLAM) family proteins CD150, CD244, and CD48. The presence of CD150 distinguishes HSC from HPC; multipotent progenitors are CD150^−^CD244^+^CD48^−^ and more committed progenitors are CD150^−^CD244^+^CD48^+^[13], though there is even variability among CD150^+^ HSC in their ability to provide balanced repopulation of irradiated bone marrow in mice [14, 15]. 

There are many limitations of phenotypic markers for identification of HSC and HPC in the context of infection and inflammation. For example, Sca-1 is upregulated on hematopoietic cells other than HSC during inflammation via interferons (INF) and tumor necrosis factor (TNF) [16, 17]. LT-HSC reduce expression of c-Kit tenfold after exposure to the chemotherapeutic agent 5-fluoruracil (5-FU) [18]. In addition, 5-FU increases expression of Mac-1 (CD11b/CD18) [18], a *β*2-integrin used by HPC for adhesion to bone marrow stroma [19] and a marker of myeloid lineage cells. Infection with an intracellular pathogen increases CD48 expression, indicative of multipotent progenitors and proliferating cells, on CD150^+^ KSL cells that would otherwise be termed LT-HSC [20]. Infection-induced stress may alter other stem and progenitor cell markers as well. Thus phenotypic strategies for detecting HSC and HPC in infection must be evaluated to avoid mistaken identification.

Regardless of the FACS methods used to isolate HSPC, functional assays are required to truly determine a cell's ability to self-renew and form mature cell lineages. In vitro assays generally measure HPC rather than primitive HSC, while long-term in vivo assays are a measure of LT-HSC. Colony-forming cell (CFC) assays determine the capacity of cells to form lineage-restricted colonies in a semi-solid, usually methylcellulose-based, media, but do not identify HSC, rather only HPC. The gold standard for differentiating LT-HSC from ST-HSC and progenitors is their ability to engraft in vivo into irradiated hosts and maintain multilineage hematopoiesis indefinitely and through serial transplantation into new hosts [21]. 

## 3. The HSPC Niche: The Bone Marrow and Beyond

As was first proposed by Schofield in 1978, HSC and HPC occupy different stem cell niches that determine cell behaviors, such as, self-renewal versus differentiation, quiescence versus proliferation, and inertia versus mobilization [22]. These behaviors are dictated by the microenvironment including physical and structural features, humoral, paracrine and neural signals, and metabolic factors. The HSPC niche most studied is the adult bone marrow, comprised of the osteoblastic and vascular niches. It is generally believed that a minority of HSC associate with endosteum and are quiescent, while a larger portion of HSC as well as HPC are located next to vascular endothelial cells and are cycling [13]. The cues received by HSPC in these environments provide a critical balance of quiescence, self-renewal, and differentiation that provides for adequate blood cells over a lifetime. 

Though HSPC primarily reside in the bone marrow, there is increasing evidence that they may find suitable niches elsewhere in the body. Indeed, it has long been recognized that extramedullary hematopoiesis (EMH), production of hemic cells outside the bone marrow, typically occurs in sites of early blood formation, such as, the liver and spleen [7]. Many tissues, however, can accommodate hematopoiesis [23] and thus may contain the essential elements of a stem cell niche. EMH has been reported in such diverse tissues as the skin, joint, urinary bladder, peritoneum, pleura, testes, adrenal glands, and gastrointestinal tract [24–31]. Myelofibrosis, inflammation, and infection are frequent underlying causes of EMH. In cutaneous pyogenic granulomas, trauma-induced vascular lesions can contain de novo formation of erythroid and myeloid cells [26, 32]. In intestinal helminth infection, multipotent progenitors cells (MPP) are induced by IL-25 in gut-associated lymphoid tissue and give rise to monocyte/macrophages and granulocytes [33]. Pleural and pericardial EMH has been associated with sepsis as well as local infections [34]. The granulocytotropic organism *Anaplasma phagocytophilum *causes mobilization of HSPC from bone marrow and enhanced myelopoiesis in the spleen [35, 36]. Thus stress, infection, and inflammation can produce stem cell niches outside of the bone marrow, which, under specific conditions, can support HSPC proliferation and differentiation. 

In the bone marrow, HSC intimately associate with osteoblasts and endothelial cells, an interaction facilitated in part by the *α*-chemokine CXCL12, also denoted stromal-derived factor-1 (SDF-1). SDF-1 is secreted by osteoblasts, stromal cells, and reticular endothelial cells and binds its receptor CXCR4 on the HSPC surface [37]. SDF-1 is the most important chemokine for maintaining HSPC within the marrow, but peripheral sites also produce this HSPC attracting chemokine. It is produced by Langerhans cells, endothelial cells, and pericytes in human skin during homeostasis and additionally by fibroblasts during inflammation [38]. SDF-1 is upregulated in many tissues secondary to ischemia and inflammation [39]. Ischemia induces SDF-1 expression by endothelial cells via hypoxia-inducible factor-1 [40]. Peripheral tissue hypoxia results in oxygen tensions similar to the bone marrow osteoblastic niche, and recruited macrophages can be supportive cells for EMH [41]. As in the bone marrow, SDF-1 gradients may play a role in the maintenance of HSPC in damaged or infected peripheral tissues.

There is a close association between HSC and endothelial cells that begins during embryogenesis [42] and remains throughout ontogeny [43]. Kiel et al. used SLAM family receptor expression to localize HSC within bone marrow and found that 60% of HSC in the bone marrow was associated with sinusoids compared to 14% associated with endosteum [13]. Their findings suggest that the endothelium expresses factors important for HSC maintenance and function. Indeed, endothelial cells in the bone marrow constitutively express adhesion molecules, such as, P-selectin, E-selectin, and vascular cell adhesion molecule-1 (VCAM-1) that are expressed by extramedullary endothelial cells during inflammation [44]. Bone marrow endothelium also elaborates hematopoietic cytokines during homeostasis, such as, stem cell factor (SCF), interleukin-6 (IL-6), granulocyte colony stimulating factor (G-CSF), and granulocyte-macrophage colony stimulating factor (GM-CSF), highlighting the role of the vascular niche in the regulation of HSC proliferation and differentiation [45]. Though bone marrow endothelium is unique in its constitutive expression of adhesion molecules and cytokines, endothelial cells from other organs can support HSPC function to varying degrees by secretion of soluble factors [46, 47]. Inflamed endothelial cells express adhesion molecules required for capture and transmigration of leukocytes and their precursors. Upon stimulation with inflammatory mediators, endothelial cells upregulate P- and E-selectins as well as adhesion molecules of the immunoglobulin superfamily, including ICAM-1 and VCAM-1 [48]. Hematopoietic growth factors are secreted by activated endothelial cells, fibroblasts, macrophages, and other innate immune cells. Interleukin-1 induces G-CSF and GM-CSF production by human umbilical endothelial cells in culture [49]. Lipopolysaccharide induces GM-CSF secretion by human pulmonary microvascular endothelial cells in vitro, and peri-tumoral inflammation leads to GM-CSF elaboration by pulmonary endothelial cells in vivo [50]. *Staphylococcus aureus *and its exotoxins induce cultured endothelial cells to secrete G-CSF as well as IL-6 and IL-8 [51]. Sites of tissue inflammation thus provide the critical constellation of cellular and soluble elements required for HSPC homing, retention, and function. 

## 4. HSPC Mobilization and Homing to Sites of Inflammation

HSPC travel through peripheral blood and lymph at low number during homeostasis, but they are mobilized by inflammation, infection, stress, and injury [4, 52–54]. Disruption of the CXCR4/SDF-1 axis results in rapid release of HSPC to the periphery [35]. In response to tissue inflammation or infection, neutrophil numbers increase in the bone marrow and release proteases, such as, matrix metalloproteinase-9 (MMP-9), neutrophil elastase, and cathepsin G [55]. These proteins act upon chemotactic and adhesion factors, such as, SDF-1, CXCR4, SCF, c-Kit, and VCAM-1, to release HSPC into circulation [54]. Additionally, cleavage fragments of the fifth complement cascade protein, C5, contribute to a proteolytic environment in the bone marrow [56]. Bacterial infection upregulates G-CSF on endothelium in the bone marrow, which inhibits osteoblast SDF-1 production and liberates HSPC from the endosteal niche [51, 57, 58]. Neural inputs to the stem cell niche also regulate HSPC mobilization. The bone marrow is innervated with noradrenergic sympathetic fibers, and HSPC express receptors for the sympathetic neurotransmitter dopamine on their surface [59, 60]. G-CSF and GM-CSF can increase neuronal receptor density on HSPC, augmenting their proliferation and motility [60]. Norepinephrine, another sympathetic neurotransmitter, suppresses osteoblast function, causing SDF-1 downregulation [61]. Thus stress works to mobilize HSPC to the periphery, where they may act in concert with other innate immune effectors for resolution of inflammation.

Homing of mobilized bone marrow HSPC to injured tissue is well documented. Hypoxic tissue, injured vasculature, and thrombi-forming platelets upregulate SDF-1 via HIF-1*α*, thus recruiting bone marrow progenitors for tissue repair [40, 62, 63]. Cleavage fragments of the third complement component (C3) increase sensitivity of CXCR4-expressing HSPC to SDF-1 gradients in injured tissue [64]. Chemokines other than SDF-1 can also attract HSPC to inflammatory sites. Monocyte chemoattractant proteins draw CCR2-expressing LT-HSC, ST-HSC and progenitor cells to injured liver and peritoneum, where they differentiate along the myeloid and lymphoid lineages [6]. Ischemia and inflammation may alter chemokine gradients between bone marrow and sites of injury to direct homing of HSPC to areas in need. 

The requirement for adhesion molecules to support HSPC homing to inflamed tissues is poorly understood. The most critical adhesion molecule for HSC maintenance in the bone marrow is VCAM-1, which binds the *α*_4_ *β*_1_ integrin very late antigen-4 (VLA-4) on the HSC surface [65–67], though the importance of this adhesion pair for HSC recruitment to nonmedullary sites is not fully defined. VLA-4 is required for HSPC entry to injured liver, whereas CD18, CD44, and PECAM-1 are not [68]. VLA-4 is also important for HSPC migration to the heart [69]. However, CD18, not VLA-4, is required for HSC homing to radiation-damaged gut [67]. Much remains to be discovered regarding the requirements for HSPC homing to infected and inflamed sites, though they are likely to include both adhesion molecules and chemokines used for BM homing as well as some that are unique to the inflamed tissue. 

## 5. HSPC Sense Infection and Inflammation**** Directly and Indirectly 

HSPC can sense infection and inflammation directly as well as indirectly via inflammatory cytokines produced by other cells. While HSC have been found to be resistant to infection by a variety of pathogens, including *Mycobacterium avium, Listeria monocytogenes, Salmonella enterica ser. typhimurium*, and *Yersinia enterocolitica* [70, 71], direct infection of HSC by pathogens, such as retroviruses, alters hematopoiesis [72]. However, intracellular infection is not the only situation in which HSPC can respond to pathogens. Ligation of HSPC pattern recognition receptors (PRR) can lead to proliferation, differentiation and elaboration of cytokines, growth factors, integrins, and receptors. Infection-induced INF signaling can cause HSC activation or inhibition depending on chronicity and context. Though HSC remain quiescent while committed progenitors supply mature leukocytes during equilibrium, they can respond rapidly during infection [73].

Interferons are mediators of inflammation, with type I INF (INF-*α* and INF-*β*) produced by virally infected cells and type II INF (INF-*γ*) produced by stimulated T cells and NK cells [73]. Toll-like receptor-3 (TLR3) recognizes viral double-stranded RNA, and injection of its ligand poly(I : C) induces type I INF, causing quiescent HSC to proliferate [74]. This response may be adaptive in viral infection but could be detrimental to maintenance of the HSC compartment with long-term exposure [74]. Although INF-*γ* inhibits hematopoiesis, maintaining homeostasis of effector cells during infection [73], several studies describe the opposite effect. Vaccinia virus infection expands the KSL bone marrow fraction and differentially regulates common myeloid progenitors (CMP) and common lymphoid progenitors (CLP) [75]. CLP differentiate to INF-*β*-producing plasmacytoid dendritic cells to combat viral infection [75]. Lymphocytic Choriomeningitis virus drives monocyte differentiation of CMP and inhibition of granulopoiesis in an INF-*γ*-dependent manner [76]. Acute malarial infection results in INF-*γ* induced c-Kit^hi^ IL-7-receptor^+^ bone marrow progenitors with both myeloid and lymphoid potential [77]. In a model of acute human monocytic ehrlichiosis, infection-induced INF-*γ* drives HSC out of dormancy and expands the KSL population in the bone marrow [20]. Interferon-*γ* likewise steers hematopoietic progenitors towards granulocyte and monocyte differentiation [20]. HSC are similarly activated in chronic infection. Persistent infection with *Mycobacterium avium* increases proliferation of KSL/Flk2^−^/CD34^−^ and SP^KLS^ LT-HSC without an increase in LT-HSC number via INF-*γ* [70]. Instead KSL/Flk2^+^/CD34^+^ST-HSC were significantly increased, suggesting compensatory proliferation of LT-HSC to supply the ST-HSC and progenitors required to meet increased peripheral leukocyte demand [70]. HSPC can sense and respond to infection indirectly via both type I and type II INF, though overstimulation of the bone marrow compartment with INF could lead to ineffective hematopoiesis and HSC exhaustion [73].

Innate immune cells can respond directly to signals of infection via PRR. PRR are signaling receptors that recognize conserved pathogen-associated molecular patterns (PAMPs) and endogenous danger-associated molecular patterns (DAMPs) to elicit immune responses. There are four main classes of PRR. These include the transmembrane toll-like receptors (TLRs) and C-type, lectin receptors (CLRs), the cytoplasmic retinoic acid-inducible gene (RIG)-I-like receptors (RLRs), and nucleotide-binding oligomerization domain or NOD-like receptors (NLRs). PRR signaling induces gene transcription leading to inflammatory responses. Recently, PRR have been identified on HSPC [8, 78–80]. Nagai et al were the first to reveal the presence of functional TLR on murine HSPC [8]. In one study, human CD34^+^ HSPC predominantly expressed transcripts for TLR1, 2, 3, 4, and 6 [81], while others have shown that TLR4, 7, and 8 are most highly expressed [80]. Additionally, the NLR NOD2 has been identified in human HSPC [78]. The expression of PRR suggests that HSPC may be an important component of the innate immune response.

When HSPC encounter microbial pathogens there is a tendency towards myeloid rather than lymphoid differentiation. TLR ligands upregulate the myeloid transcription factors PU.1, C/EBPa, and GATA-1 on HSPC, directing lineage fate decisions [81]. The NLR NOD2 in human CD34^+^ HSPC is activated by the bacterial peptidoglycan muramyl dipeptide (MDP) resulting in increased expression of PU.1, a transcription factor important in driving hematopoietic progenitor lineage fate [80]. NOD2-stimulated HSPC differentiate to dendritic cells and macrophages. Upon ligand binding in vitro, both TLR2 and TLR4 expressed on HSPC induce proliferation and differentiation to monocytes and dendritic cells in the absence of hematopoietic growth factors [8]. *Candida albicans* yeast and hyphae are signals through HSPC TLR2 to produce phagocytic macrophages and neutrophils in vitro [82, 83]. In an in vivo mouse model of invasive candidiasis, hematopoiesis was skewed towards granulocytes and monocytes, with a corresponding decrease in B-cell formation [83]. Thus, upon detection of PAMPs, HSPC preferentially differentiate towards mature myeloid immune cells to fight infection.

## 6. HSPC as Immune Effectors

Stem and progenitor cell function in peripheral sites of inflammation has only recently been reported. Circulating HSPC may act as sentinels of infection, serve as readily available precursors of mature blood leukocytes, or participate as effectors themselves. HSPC respond to and secrete chemokines, pro- and anti-inflammatory cytokines and growth factors [84, 85]. For example, human CD34^+^ cells secrete SCF, FLT3-ligand, insulin-like growth factor-1, thrombopoietin, and transforming growth factor-*β*1 (TGF-*β*1) and TGF-*β*2, which have variable effects on HSC viability [84]. Conditioned media from CD34^+^ cells was a chemoattractant and increased survival and proliferation of other CD34^+^ cells [84]. After stimulation with *S. aureus* CD34^+^ HSPC produced high levels of TNF, IL-6, IL-8, IL-23, and IL-10 [85]. Upon ligation of NOD2 by MDP, human CD34^+^ cells expressed TNF*α*, IL-1*β*, and GM-CSF [78]. They also upregulated intracellular stores of *α*-defensins 1–3, suggesting that they may be able to directly fight bacterial infections [78]. There appears to be synergism between NOD2 and TLR signaling, as cytokine production is significantly elevated when HSPC are stimulated with NOD2 and TLR ligands together versus individually [78]. Infection indirectly stimulates HSPC to generate reparative growth factors, including TGF-*β*, epithelial growth factor, angiogenin, fibroblast growth factor, platelet-derived growth factor, SDF-1, and vascular endothelial growth factor [85]. CD34^+^ human HSPC can produce Th2 cytokines, like IL-5, IL-13, IL-6, and GM-CSF, at levels up to 100-fold greater than activated mast cells [85, 86]. In a mouse model of intestinal helminth infection, c-Kit^+^/lineage^−^ multipotent hematopoietic progenitor cells trafficked to gut-associated lymphoid tissue where they secreted Th2 cytokines [33]. The resulting progeny provided protective immunity when transferred to mice susceptible to helminth infection. Thus HSPC can secrete inflammatory cytokines, chemokines, growth factors, and antimicrobial peptides and may participate in the response to invading pathogens.

HSPC can directly respond to infection in the periphery. The seminal work of Massberg et al. illustrated not only the roaming nature of HSPC in blood and lymph but also the ability of LPS to activate HSPC injected into the renal subcapsular space to produce myeloid and dendritic cells [3]. More recently, Kim et. al. demonstrated that lineage-negative c-Kit^+^ HSPC traffic to *Staphylococcus aureus*-infected skin wounds and proliferate and differentiate locally to mature neutrophils [5]. This response is mediated in part by TLR2 and MyD88 [87]. Schmid et al. demonstrated that common dendritic cell progenitors in the bone marrow express TLR2, 4, and 9 and, upon activation, downregulated CXCR4 [88]. These common dendritic cell progenitors preferentially trafficked to inflamed lymph nodes and gave rise to dendritic cells [88]. HSPC act in the periphery as a source of mature effectors, providing for immediate local leukocyte needs, and as innate immune cells, elaborating inflammatory responses and mediating tissue repair. 

## 7. Future Directions

HSPC can respond to infection and inflammation by expansion in the bone marrow, mobilization to the circulation and peripheral tissue, proliferation, differentiation, and elaboration of secreted factors. HSPC thus may fine-tune the innate immune response. It is unknown whether peripheral HSPC responses influence the outcome of inflammatory or infectious diseases. As one arm of innate defense, the roles of HSPC could be beneficial or detrimental. Indeed, HSPC may contribute to maintenance of aberrant inflammation in allergy and asthma [89]. HSPC may alter the phenotype of other immune cells. For example, HSPC may direct a proinflammatory versus anti-inflammatory macrophage phenotype or a Th1 versus Th2 T-cell response.

In order to fully dissect HSPC function, it will be critical to understand the mechanisms for HSPC homing to infected tissue. There is evidence that the chemotactic signals and adhesion molecules used by HSPC to recruit to inflamed vessels are tissue specific. Thus it may be conceivable to prevent or augment HSPC entry into a particular organ of interest. One significant challenge in this endeavor is that HSPC share chemokine receptors and adhesion molecules with other leukocytes, thus blocking one cell type would affect others. A novel approach for more specifically manipulating HSPC trafficking is needed. 

It is likely that the HSPC response to infection will depend on the pathogen, the tissue, and the infectious burden. The magnitude and type of HSPC activation during sepsis versus localized infection will likely diverge. It will be important to determine if effector cells produced de novo by HSPC at sites of inflammation are functionally distinct from leukocytes entering these sites from peripheral blood. HSPC expansion within infected tissue could be doing more than simply providing mature leukocytes, but rather they could be orchestrating the local innate immune response. Exploiting the HSPC response might provide a novel means of biological therapy in an era of increasing antibiotic-resistant infections. A deeper understanding of the mechanisms of HSPC mobilization, recruitment, and activation could provide the tools necessary to manipulate them for endogenous cell-based therapy.

## 8. Conclusions

Mounting evidence suggests that HSPC are not simply a source of leukocytes in the bone marrow but are active players in the innate immune response to local and systemic insults. As more studies employ population-specific phenotyping and functional verification of those populations, the identification of specific HSC and HPC effects in the response to infection will become increasingly clear. HSPC produce mature effector cells in the bone marrow upon sensing danger signals from the periphery. That HSPC can traffic to sites of inflammation outside of the bone marrow suggests that they are playing a relevant within these sites. They may combat infection on the front line by providing reconnaissance, peripheral hematopoiesis, and battling pathogens via elaboration of soluble mediators of inflammation. Further exploration of the role of HSPC in innate immunity may provide valuable tools for fighting infection.
